# Nanometre-thick single-crystalline nanosheets grown at the water–air interface

**DOI:** 10.1038/ncomms10444

**Published:** 2016-01-20

**Authors:** Fei Wang, Jung-Hun Seo, Guangfu Luo, Matthew B. Starr, Zhaodong Li, Dalong Geng, Xin Yin, Shaoyang Wang, Douglas G. Fraser, Dane Morgan, Zhenqiang Ma, Xudong Wang

**Affiliations:** 1 Department of Material Science and Engineering, University of Wisconsin—Madison, 1509 University Avenue, Madison, Wisconsin 53706, USA; 2 Department of Electrical and Computer Engineering, University of Wisconsin—Madison, Madison, Wisconsin 53706, USA

## Abstract

To date, the preparation of free-standing 2D nanomaterials has been largely limited to the exfoliation of van der Waals solids. The lack of a robust mechanism for the bottom-up synthesis of 2D nanomaterials from non-layered materials has become an obstacle to further explore the physical properties and advanced applications of 2D nanomaterials. Here we demonstrate that surfactant monolayers can serve as soft templates guiding the nucleation and growth of 2D nanomaterials in large area beyond the limitation of van der Waals solids. One- to 2-nm-thick, single-crystalline free-standing ZnO nanosheets with sizes up to tens of micrometres are synthesized at the water–air interface. In this process, the packing density of surfactant monolayers adapts to the sub-phase metal ions and guides the epitaxial growth of nanosheets. It is thus named adaptive ionic layer epitaxy (AILE). The electronic properties of ZnO nanosheets and AILE of other materials are also investigated.

## Abstract (web summary)

The recently discovered phenomena arising from 2D nanomaterials have led to an increased interest in the fabrication of other ultrathin materials from those typically only observed in the bulk. Here, the authors demonstrate the synthesis of micron-sized, single-crystalline ZnO nanosheets via solution based methods.

## Graphical abstract

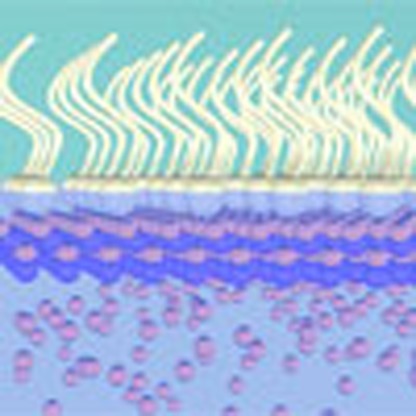

Two-dimensional (2D) nanomaterials, in particular when their thickness is just one or a few atomic layers, exhibit physical properties dissimilar to those of their bulk counterparts and other forms of nanostructures. Graphene and transition metal dichalcogenides have epitomized the applications of 2D nanostructures in many electronic, optoelectronic and electrochemical devices^1^,^2^,^3^,^4^,^5^,^6^,^7^. Nonetheless, real-world 2D nanostructures so far have been largely limited to naturally layered materials, that is, the van der Waals solids, synthesized either from top-down or bottom-up^8^,^9^,^10^. A much larger and diverse portfolio of 2D materials including non-layered compounds are desirable to meet the specific requirements of individual components in various devices^11^. When seeking a synthesis route to create nanosheets from non-layered materials, it is critical to break the crystal symmetry and foster the anisotropy in crystal growth. Unlike one-dimensional nanostructures whose growth mechanisms have been well described, syntheses of ultrathin 2D nanomaterials from non-layer materials without an epitaxial substrate have remained a case-by-case practice^12^. In the sporadic literature reports, such as PbS nanosheets by oriented attachment and Pd nanosheets by a solvothermal method^13^,^14^,^15^, the size of the nanosheets is usually below 1 μm and much smaller than that of graphene and transition metal dichalcogenides, which presents myriad fabrication challenges for real devices. In this regard, a novel and yet robust synthesis strategy dedicated to the growth of general 2D nanostructures of large sizes would enable many novel materials to be grown at a practical dimension for applications.

To control the morphology of nanomaterials in solution synthesis, one of the most common strategies is to employ surfactant molecules or inorganic ions that are preferentially adsorbed on specific crystallographic facets, the growth of which is then retarded and thus the growth of other facets are promoted^16^,^17^. One intriguing strategy is to use surfactant monolayer at the water–air interface as a soft template, to guide the growth of nanostructures underneath. By using an arachidic acid monolayer as templates, oriented, sub-100-nm-sized PbS nanoplates and nanorods were obtained when the surface pressure of the monolayers were optimized^18^,^19^,^20^. We hypothesize that once a strong recognition between the surfactant monolayer and the target materials in the aqueous sub-phase was established, epitaxial growth could be realized over a large area. In our prior work, a few hundred nanometre-thick, micrometre-sized zinc hydroxyl dodecylsulfate nanomembrane laminates were synthesized at the water–air interface by introducing high-concentration sodium dodecyl sulfate to the ZnO growth solution^21^,^22^, revealing the possibility of using an anionic sulfate monolayer to induce the growth of single-crystalline ZnO nanosheets with large sizes. Here we report that ∼1- to 2nm-thick single-crystalline ZnO nanosheets with sizes up to tens of micrometres can be synthesized at the water–air interface.

## Results

### Morphology and structural characterization of ZnO nanosheets

**Figure 1:**
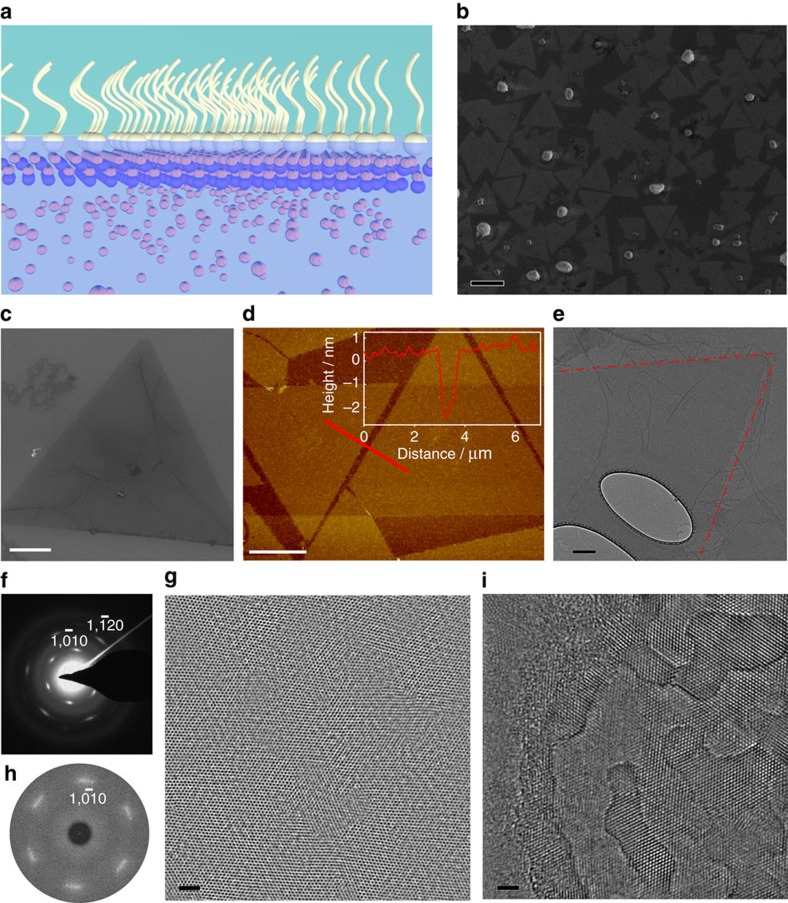
Morphology of ZnO nanosheets. (**a**) Schematic illustration of the formation of ZnO nanosheets directed by surfactant monolayer. (**b**) Scanning electron microscopy (SEM) image of the nanosheets on a silicon substrate coated with 100 nm SiO_2_. Scale bar, 10 μm. (**c**) SEM image showing a typical nanosheet with an equiangular triangle shape. Scale bar, 5 μm. (**d**) Atomic force microscopy (AFM) topography scans of typical nanosheets with flat surfaces on a Si substrate. Scale bar, 5 μm. (**e**) TEM image of a corner of a 20-μm-sized ZnO nanosheet. Scale bar, 200 nm. (**f**) Corresponding SAED pattern of the nanosheet shown in **e**. (**g**) HRTEM image of the same nanosheet. Scale bar, 2 nm. (**h**) Corresponding Fourier transformation that shows a hexagonal symmetry. (**i**) HRTEM image of a nanosheet showing overlayer growth. Scale bar, 2 nm.

In this synthesis, oleylsulfate anionic monolayers were employed to guide the growth of ∼1- to 2-nm-thick single-crystalline ZnO nanosheets at the water–air interface. Sodium oleylsulfate was first dissolved in chloroform and subsequently spread over the surface of an aqueous solution containing precursors that would otherwise produce chunky ZnO nanocrystals (see Methods). Although ZnO nanocrystals still form in the bulk part of the solution, there appeared a single layer of ZnO nanosheets that covered the entire water–air interface. As schematically shown in Fig. 1a, the oleylsulfate anions form a close-paced monolayer at the water–air interface, under which Zn^2+^ cations are supersaturated and precipitate into nanosheets. Much similar to graphene, when the nanosheets were transferred onto oxide-coated Si substrates, they became visible under the optical microscope and this was very useful for subsequent characterization and device fabrication (see Supplementary Fig. 1a)^23^. Figure 1b is a scanning electron microscopy image of ZnO nanosheets on a 100-nm SiO_2_-coated Si substrate. These nanosheets were densely packed and nearly covered the entire surface (see Supplementary Fig. 2b,c). A small number of nanoparticles were found sparsely distributed in the nanosheet layer. We suspect that the formation of these nanoparticles is due to the precursors being dissolved in the residue chloroform that is used to disperse the monolayer (see Supplementary Discussion). Figure 1c shows a single triangular nanosheet with edges longer than 20 μm. This is the typical morphology of the as-received nanosheets. A topography atomic force microscopy scan revealed the nanosheet was 2.28 nm in thickness and nearly uniform across the entire area (Fig. 1d). The surface roughness was found to be 0.2 nm. Because of the way we transferred the nanosheets to substrates, an oleylsurfate monolayer was inevitably transferred along with the nanosheets. X-ray photoelectron spectroscopy characterization of the nanosheets confirmed the presence of oleylsulfate on the surface of nanosheets and the specific bonding between sulfate groups and Zn^2+^ ions (see Supplementary Note 1.

Transmission electron microscopy (TEM) was applied to investigate the crystallinity of these nanosheets and their formation mechanism. The size of the nanosheet was too large to be fully imaged (see Supplementary Fig. 3). Figure 1e shows a corner of a triangular nanosheet, which is slightly darker in contrast compared with the background due to its ultra-small thickness. For a clear presentation, we highlighted the edges with red dashed lines. Several very thin whiskers were observed as well, which might be concentrated surfactant residues. Selective area electron diffraction (SAED) pattern revealed a single-crystalline hexagonal lattice with a *d*-spacing of 0.281 nm, which matches the Wurtzite ZnO (0001) facet (Fig. 1f). High-resolution (HR) TEM images were obtained on the area through the holes of the holey-carbon film, as shown in Fig. 1g. The HRTEM revealed the single-crystalline nature of the nanosheet, whereas dislocations or small defective areas could also be observed on the nanosheet. Corresponding fast Fourier transfer (FFT) pattern of the HRTEM image clearly matches the SAED pattern, confirming the single crystallinity across the entire nanosheet (Fig. 1h). Nanosheets with uneven surfaces were also observed as shown in Fig. 1i. There appeared to be a developing overlayer on the nanosheet surface, indicative of a layer-by-layer growth mode.

### Time evolution of ZnO nanosheets

**Figure 2:**
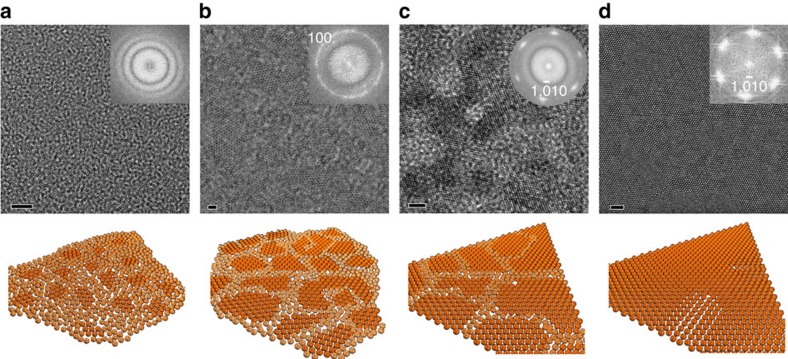
TEM images and schematic drawings showing the time-dependent evolution of ZnO nanosheets. (**a**) Mostly amorphous films with tiny crystalline grains and curved edges. (**b**) More crystallized nanosheets with 2–3 nm grains that are randomly oriented. (**c**) These crystallized grains grew larger and had aligned orientation. (**d**) Large-area single-crystalline nanosheet. The insets are FFT patterns of the TEM images, respectively. The four schematic drawings below TEM images conceptually depict the crystal structure of each stage during the evolution of ZnO nanosheets. Regions with lighter gold-coloured spheres are amorphous and regions with deeper gold-coloured spheres are crystallized. Scale bars, 5, 2, 2 and 2 nm, respectively.

The nanosheets at the water–air interface were collected at different reaction times to investigate their formation mechanism. TEM images in Fig. 2a–d and the conceptual drawing below them respectively illustrate the crystal structure evolution of the nanosheets. What appeared at the interface first was a continuous amorphous film (Fig. 2a), which is supported by the inset FFT pattern. Tiny crystallites are embedded in the largely amorphous film although hardly visible. These crystallites then grew in lateral size and were all oriented with the same hexagonal crystal plane exposed; however, their in-plane rotation appeared to be stochastic, as shown in Fig. 2b. The inset FFT pattern confirmed such a textured structure, with a single ring that matches the *d*-spacing of the SAED pattern. As the crystallites grew larger, they merged at an aligned orientation into a contiguous, single-crystalline network coexisting with much reduced amorphous region confined between the nanosheets (Fig. 2c). A single set of sixfold symmetric spots appeared in the FFT pattern of the TEM image. Eventually, the amorphous area was fully crystallized and the nanosheet became single crystalline with few dislocations that were probably formed by the mis-orientation of merged crystalline areas during the formation process (Fig. 2d).

### Zn^2+^ concentration under the ionic monolayer

Considering the formation of ZnO from solution was driven by Zn^2+^ concentration, we simulated the effects of a negatively charged surfactant monolayer on the concentration profile of Zn^2+^ near the water–air interface, to understand the origin of the nanometre-level thickness. We assumed the average area occupied by a single oleylsulfate molecule in the monolayer is 0.2 nm^2^, which corresponded to a charge density of −0.801 C m^−2^. A positively charged Stern layer with a uniform charge density of 0.736 C m^−2^ was established at a distance of 0.30 nm away from the surfactant layer (see Supplementary Note 2). The electric field resultant from superimposing both charged surfactants and Stern layers influences the charged species in solution, attracting (concentrating) or repulsing (diluting) positively and negatively charged ions, respectively. The electrical potential profile, (*ϕ*(*x*)), between the Stern layer and bulk solution was solved numerically using the following equation^24^:


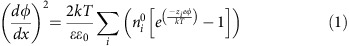


where *ϕ* is the potential relative to bulk solution, *x* is a measure of distance into solution and perpendicular to the monolayer surface, *k* is the Boltzmann constant, *T* is absolute temperature, 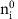 is the bulk concentration of ion *i*, *z*_*i*_ is the charge of ion *i* and *e* is the charge of an electron. The potential adjacent to the Stern layer at the closest approach by ions in solution, *ϕ*(6 Å), was taken as 0.065 V, which corresponds to the closest packing of ions in solution (Supplementary Fig. 8). As the concentrations of all species are constantly changing during the reaction, we calculated the chemical environment near the interface only at the relevant reaction time point (1.5 h into the reaction) assuming the concentration of each ion as listed in Supplementary Table 1. Consistent with equation (1), the ratio of concentrations of species *i* in bulk solution 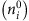 at bulk solution potential (*ϕ*^0^=0) to its concentration (*n*_i_) found at any other potential (*ϕ*) is given by^24^:


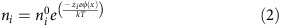


**Figure 3:**
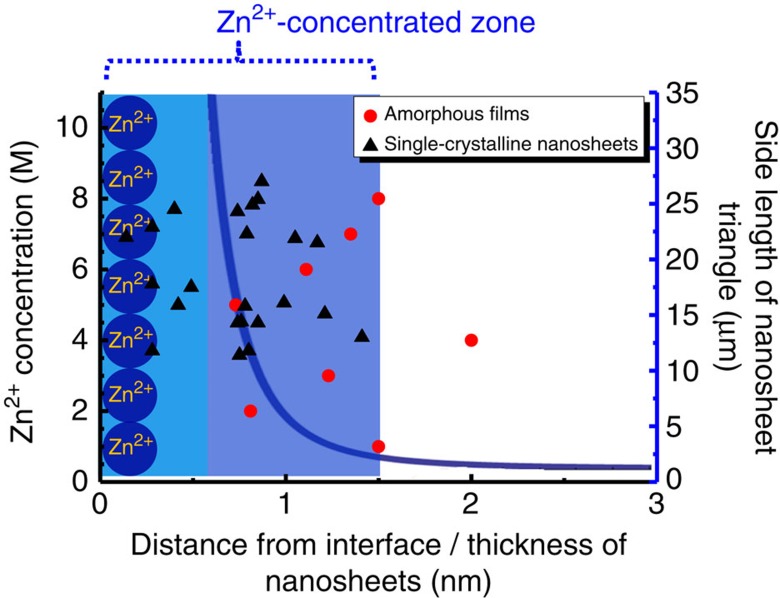
Thickness and size relation to Zn^2+^ concentration distribution. The blue shaded band represents a positively charged stern layer primarily composed of Zn^2+^ ions. The blue curve plots the concentration of Zn^2+^ from the end of the Stern layer (blue shaded area within the first 0.6 nm) into the bulk solution. The black triangles and red round dots marks the thickness of monocrystalline nanosheets and amorphous nanosheets formed prior, respectively, measured by atomic force microscopy (AFM). The right vertical axis is the side length of single-crystalline nanosheet triangles. It is noteworthy that the size of amorphous nanosheets are hundreds of micrometres and are not displayed in this plot.

In the case of Zn^2+^, 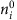 is 18.72 mM and *z*_i_ is 2. Figure 3 presents a plot of Zn^2+^ concentration as a function of distance from the surfactant monolayer into the bulk solution. It can be observed that the concentration of Zn^2+^ ions dropped drastically within a very short distance from the Stern layer. At distant *x*=1.5 nm, its concentration became only marginally different from bulk concentration. Therefore, the Stern layer and the diffuse Gouy layer up to 1.5 nm from the interface constituted a Zn^2+^-concentrated zone. In Fig. 3, we also marked the measured thickness of the single-crystalline nanosheets and the amorphous films by black triangles and red round dots, respectively (see Supplementary Fig. 5). The single-crystalline triangular ZnO nanosheets and amorphous films have an average thickness of 2.84±0.26 and 3.28±0.41 nm, respectively. The single-crystalline ZnO nanosheets were generally thinner than the amorphous films, possibly due to a volume reduction and removal of non-native ions (for example, nitrate and ammonium ions) during the crystallization. Given the fact that the oleylsulfate adsorbed on the surface of nanosheets may have contributed a thickness of ∼2 nm (refs 25), the thickness of the amourphous ZnO nanosheets is about the same as the width of the Zn^2+^-concentrated zone near the interface (∼1.5 nm). We therefore propose that this Zn^2+^-concentrated zone has provided an interfacial chemical environment different from the bulk concentration, which drove the growth of ZnO nanosheets, and was directly related to the thickness of the initial amorphous ZnO films that subsequently transformed into single-crystalline nanosheets. We also found that the thickness of the nanosheets increased by increasing the density of the oleylsulfate monolayers (see Supplementary Note 4). With a denser anionic monolayer, there would be more Zn^2+^ cations in the Stern layer to screen the electric field formed by the anionic monolayer, providing more precursors for the growth of nanosheets. Therefore, the nanosheets formed in the Zn^2+^-concentrated zone were thicker.

### Electrical properties

**Figure 4:**
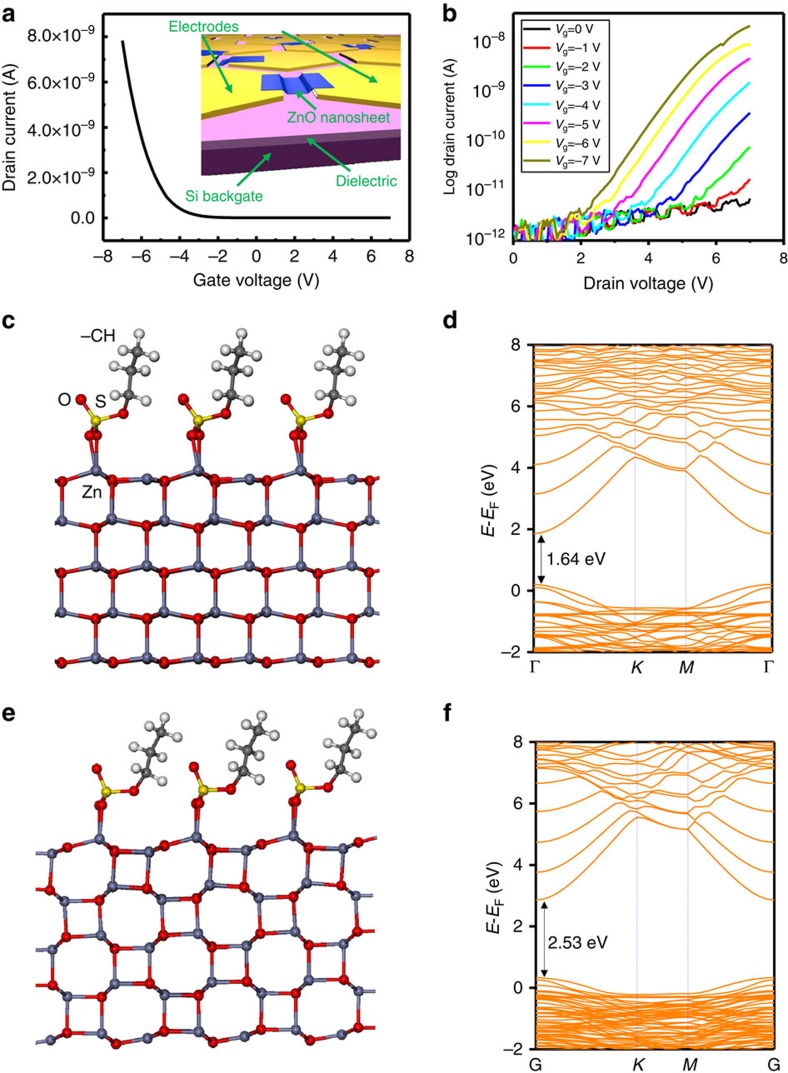
Electronic properties of ZnO nanosheets. (**a**) Drain current versus gate voltage when the drain voltage is 5 V. The gate voltage scan was from 7 to −7 V. (**b**) Drain current versus drain voltage at different gate voltages from 2 to −7 V with a 1 V step. (**c**,**d**) Simulated molecular structure and electronic band structure of wurtzite ZnO nanosheets with surfactant molecules on the surfaces. (**e**,**f**) Simulated molecular structure and electronic band straucture of tetragonal ZnO nanosheets with surfactant molecules on the surfaces. In **d** and **f**, Γ=(0, 0, 0), *K*=(−1/3, 2/3, 0) and *M*=(0, 1/2, 0).

The electronic property of as-synthesized ZnO nanosheets were further investigated by fabricating thin film transistors with a back gate configuration (inset of Fig. 4a) (see Methods for details in device fabrication). The *I*_d_−*V*_g_ curve shows an increasing source-drain current (*I*_d_) as the gate voltage (*V*_g_) scans from positive to negative (Fig. 4a). This is a typical *p*-type semiconductor behaviour. The *I*_d_−*V*_d_ curves at different gate voltages shown in Fig. 4b further confirmed the *p*-type conductivity of the ZnO nanosheets. Higher positive drain current was obtained as the gate voltage went more negative. Based on the dimension of the nanosheets and the transconductance derived from Fig. 4a, the carrier concentration and hole mobility of the nanosheets were estimated to be 4.5 × 10^12^ cm^−2^ and 0.10 cm^2^ V s^−1^, respectively (see Supplementary Methods). The low mobility might be a result of poor electrical contact between the nanosheet and electrodes due to the presence of surface surfactants. We fabricated and measured 20 devices, and carrier concentration and hole mobility values were all within the same order of magnitude.

No matter in the form of bulk crystal, thin films or nanostructures, ZnO is a wide band gap semiconductor and typically exhibits strong *n*-type conductivity due to native defects. Intentional *p*-type doping of ZnO has been a contentious topic, although few successful doping strategies have yielded fairly stable *p*-type conductivity^26^,^27^,^28^,^29^. Given the few nanometre thickness, it is reasonable to assume the surface adsorbed molecules might have had profound influence on the electrical properties^30^,^31^. To further understand the atomistic structures and electronic properties of the 2D ZnO, we theoretically investigate a ∼1-nm-thick ZnO(0001) slab through density functional theory calculations (see Supplementary Methods) using Heyd–Scuseria–Ernzerhof functional. We consider three possible structures: the wurtzite structure; a planar or graphite-like structure, which was theoretically proposed for thin ZnO(0001) slabs^32^; and a tetragonal structure, whose bulk form possess a P42/MNM symmetry (space group number: 136) and was first discovered theoretically in ZnO nanorods under high pressure (see Supplementary Fig. 7)^33^. Both wurtzite and tetragonal structures were found stable in the form of nanosheet with surfactants attached on the (0001) surface, whereas the tetragonal phase might be slightly more energetically favourable (see Supplementary Note 2). The simulated structure of wurtzite and tetragonal ZnO nanosheets and corresponding electronic band structures are shown in Fig. 4c–f, respectively. We note that as the tetragonal structure has a similar hexagonal symmetry as wurtzite ZnO (0001) along the normal direction of the nanosheets (*c* axis in a tetragonal lattice), SAED patterns of the nanosheets could not distinguish between the two phases (see Supplementary Fig. 9). The band structures in Fig. 4d,f show that the two possible structures are both direct bandgap *p*-type semiconductors. Although the Heyd–Scuseria–Ernzerhof functional^34^ used here may underestimate the band gap, the qualitative determination on the type of semiconductor found here is unlikely to be changed with more advanced calculations.

## Discussion

Although phase transformations from amorphous films to single-crystalline nanosheets have been seen in literature^35^,^36^, the transformation to ultrathin 2D nanostructures in the scale of tens of micrometres is unprecedented. We believe that the densely packed anionic monolayer of oleylsulfates at the water–air interface has not only stimulated the growth of large, amorphous ZnO films but has also directed the crystallization process of ZnO nanosheets across a large 2D scale. This multistep process began when the anionic monolayer created a negative surface potential that raised the concentration of Zn^2+^ near the monolayer, resulting in the initial formation of the amorphous films described in Fig. 2a. As the amorphous ZnO film began to crystallize, the individual ZnO crystallites would align themselves and merge into larger crystalline nanosheets. The driving force of such a long-range self-alignment is expected to be the combined effect of the strong association between the oleylsulfate headgroups and the Zn^2+^ ions below, and the van der Waals interaction among the hydrocarbon tails. To test this hypothesis, control experiments were conducted using stearic acid in place of oleylsulfate, as it has a similar molecular structure but a different headgroup (carboxylate). Millimetre-sized, nanocrystal-percolated amorphous films without faceted edges were obtained, very similar to the initial amorphous films when oleylsulfate was used (see Supplementary Note 3). No single-crystalline nanosheet triangles were observed after extended reaction time. This corroborated our argument that specific bonding between sulfate groups and ZnO surface are needed for the crystallization of ZnO nanosheets. Large, single-crystalline nanosheets were also obtained with great reproducibility under oleylsulfate monolayers with 1.5 × and 2 × density (see Supplementary Note 4). This indicates that single-crystalline ZnO nanosheets grew under the surfactant monolayer in a different way than conventional epitaxy growth where the lattice parameters must match those of the growing material. We believe this is because the Zn^2+^ ions imposed a profound influence on the arrangement of the anionic surfactant monolayer, as metal ions in the aqueous sub-phase, especially multivalent ones, can affect the 2D arrangement of surfactant molecules in monolayers and improve their stability via electrostatic and coordination interactions^37^,^38^. Therefore, during the formation of ZnO nanosheets, the local packing density of the oleylsulfate anions can spontaneously and simultaneously adapt to the ZnO lattice. This growth therefore occurs through a two-way epitaxy process and is thus named as adaptive ionic layer epitaxy (AILE). Based on this mechanism, we argue that AILE could be broadly applied to synthesizing 2D nanosheets from a wide range of materials. By designing appropriate combination of anionic surfactant monolayer and metal ion solution, large area nanosheets were synthesized from NiO and Au as well (see Supplementary Note 5). Although their crystallinity and thickness still need to be further optimized, the success of initial syntheses demonstrated potentials of AILE in creating 2D nanomaterials from non-van der Waals solids. When it comes to the electrical properties of nanosheets, regardless of the crystal structures of the ZnO slab, our simulation results demonstrate that the species of the surfactants used to form the monolayers can significantly affect the electrical properties of the grown materials, showcasing the power of AILE as a novel synthesis method for tuning the physical properties of nanosheets.

In summary, we have developed a solution-based technique to synthesize large-area, single-crystalline nanosheets guided by surfactant monolayers. Around 1- to 2-nm-thick, tens of micrometre-sized ZnO nanosheets were obtained. The universality of this technique was further demonstrated by the syntheses of other oxide and metal nanosheets. Our calculation results established a correlation between the Zn^2+^-concentrated zone under the surfactant monolayer and the thickness of the nanosheets. Simulation of band structure of oleylsulfate-adsorbed ZnO nanosheets was performed to explore the origin of the *p*-type conductivity observed experimentally. This AILE technique, with similar attributes in the processes found in biomineralization, shows great promises as a novel and versatile synthesis paradigm for forming nanosheets from a wide range of inorganic materials including and beyond the van der Waals solids.

## Methods

### Synthesis of ZnO nanosheets

In a typical synthesis, 17 ml aqueous solution containing 25 mM Zn(NO_3_)_2_ and hexamethylenetetramine was prepared in a glass vial. Subsequently, 10 μl chloroform solution containing 0.1 vol % sodium oleyl sulfate was spread on the water surface. This glass vial was then screw-capped and placed in a 60 °C convection oven. ZnO nanosheets would appear in ∼1 h 40 min and could be scooped using an arbitrary substrate for characterization and device fabrication.

### Fabrication process of ZnO nanosheet-based field-effect transistors

A 50-nm-thick layer of Al_2_O_3_ by atomic layer deposition at 300 °C was first coated over a heavily doped Si substrate. A few 300 mesh Cu TEM grids (bar width of 10 μm) were then attached onto each substrate. These TEM grids were used as shadow masks and the substrate was coated with Cr/Au/Cr (5 nm/45 nm/5 nm) by e-beam evaporation. After removing the grids and thoroughly cleaning the substrates with isopropanol and acetone, the substrates were used to directly scoop the nanosheets from the surface of the reaction solution. As the nanosheets were densely distributed on the water–air interface, some of them naturally sat between two hexagonal metal pads defined by TEM grids. These nanosheet-based field-effect transistors were then measured by probes without any further treatment.

## Additional information

**How to cite this article:** Wang, F. *et al.* Nanometre-thick single-crystalline nanosheets grown at the water–air interface. *Nat. Commun.* 7:10444 doi: 10.1038/ncomms10444 (2016).

## Supplementary Material

Supplementary Information Supplementary Figures 1-12, Supplementary Tables 1-2, Supplementary Notes 1-5, Supplementary Discussion, Supplementary Methods and Supplementary References
