# Supplementary material for: Nanometre-thick single-crystalline nanosheets grown at the water–air interface
Source: Nat Commun. 2016 Jan 20;7:10444. doi: 10.1038/ncomms10444 (PMC4736115; doi:10.1038/ncomms10444)
Supplement: Supplementary Information — Supplementary Figures 1-12, Supplementary Tables 1-2, Supplementary Notes 1-5, Supplementary Discussion, Supplementary Methods and Supplementary References [file ncomms10444-s1.pdf]

Supplementary Figures

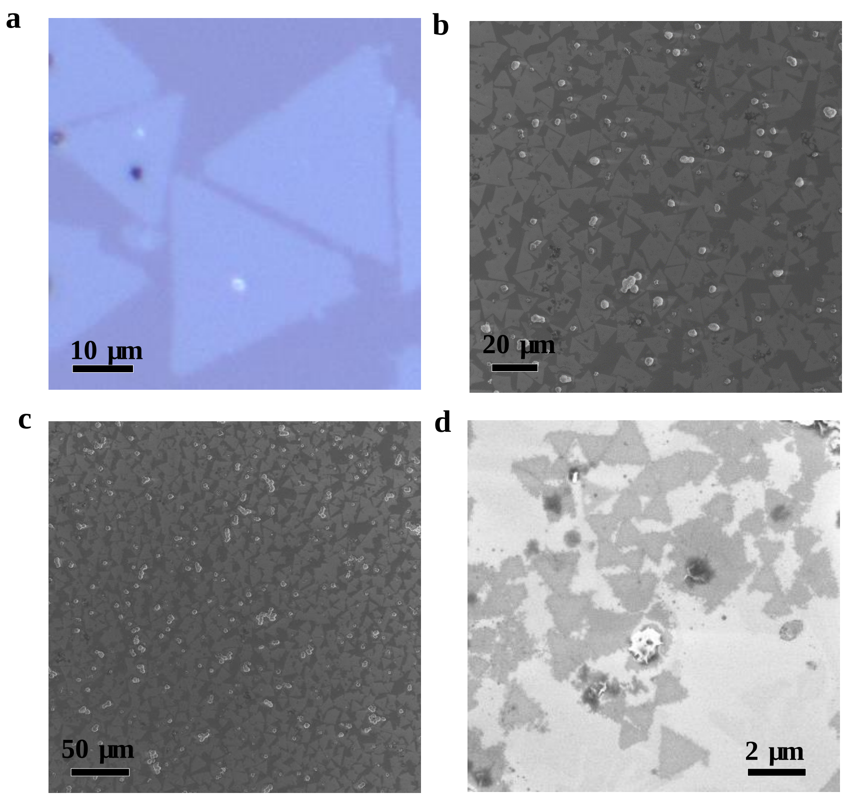

Supplementary Figure 1. Additional of single-crystalline ZnO nanosheet triangles densely transferred to a SiO2-coated Si substrate. **a**. Optical microscopy image, **b** & **c**, low magnification SEM images. The nanosheets show lighter contrast. **d**, SEM image of nanosheets synthesized at water-cyclohexane interface.

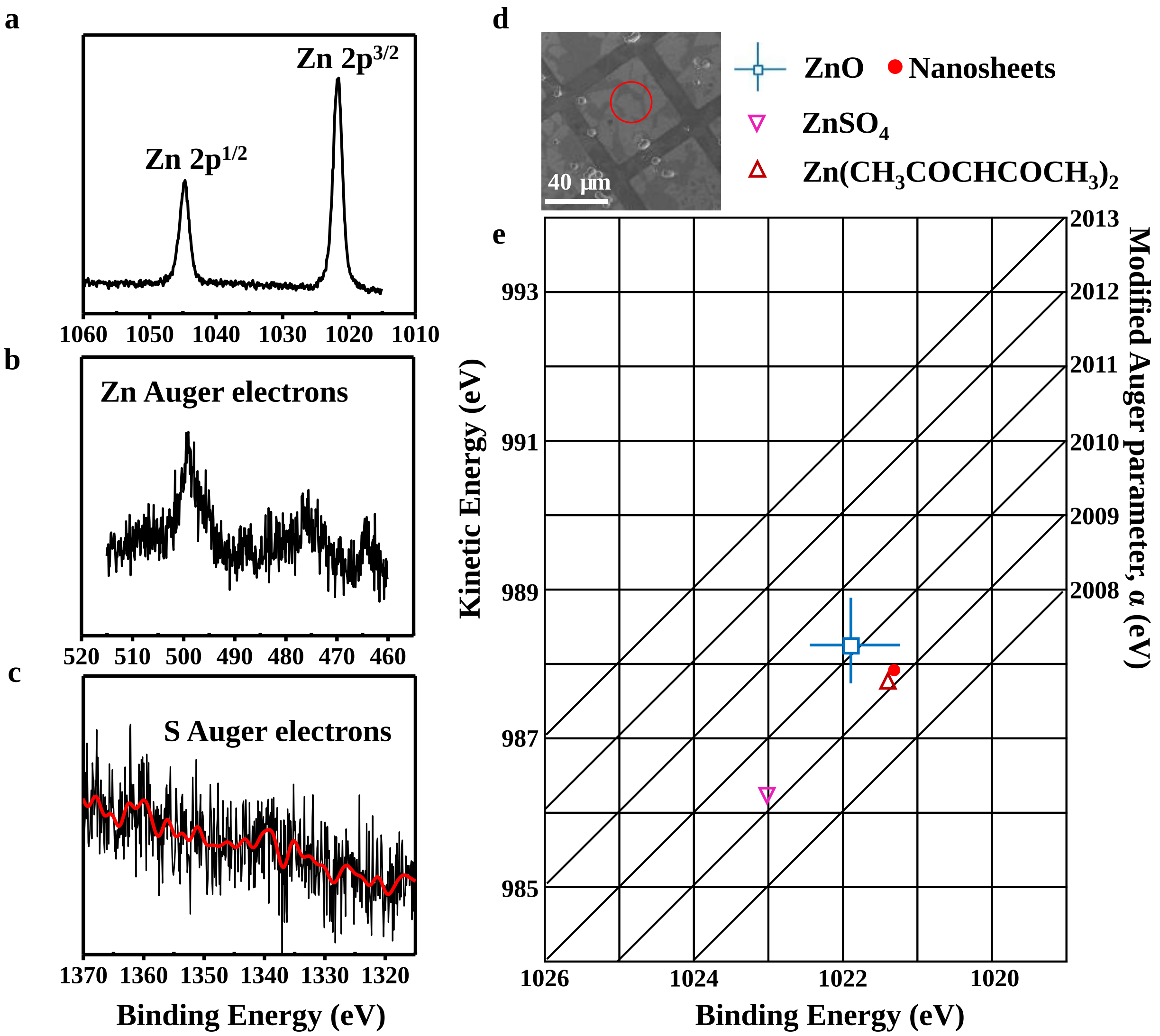

Supplementary Figure 2. | XPS characterization of ZnO nanosheets. a, XPS spectrum of Zn. **b, and c,** Auger electron spectra of Zn and S, respectively. The red curve in **c** is a smoothed curve of the spectrum. **d,** SEM image of the nanosheet that was focused on to acquire the XPS spectra. **e,** Wagner plot of Zn based on the binding energy and kinetic energy of Zn.

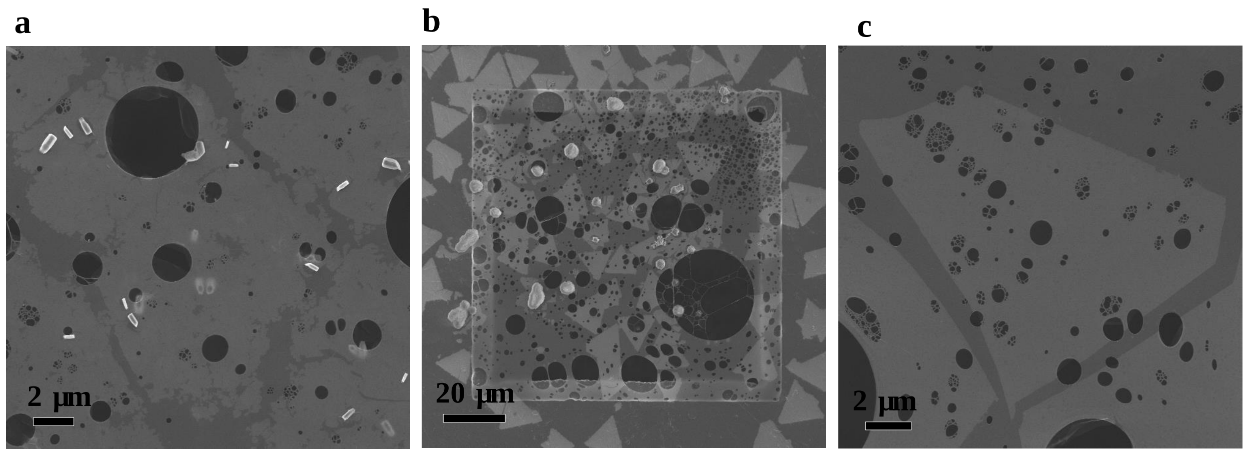

Supplementary Figure 3. | SEM images of ZnO triangle nanosheets on a holey carbon film TEM grid. a, Polycrystalline nanosheets without faceted edges obtained at earlier stage of the reaction. **b, and c,** Low-magnification and zoomed-in images of single-crystalline ZnO triangle nanosheets, respectively.

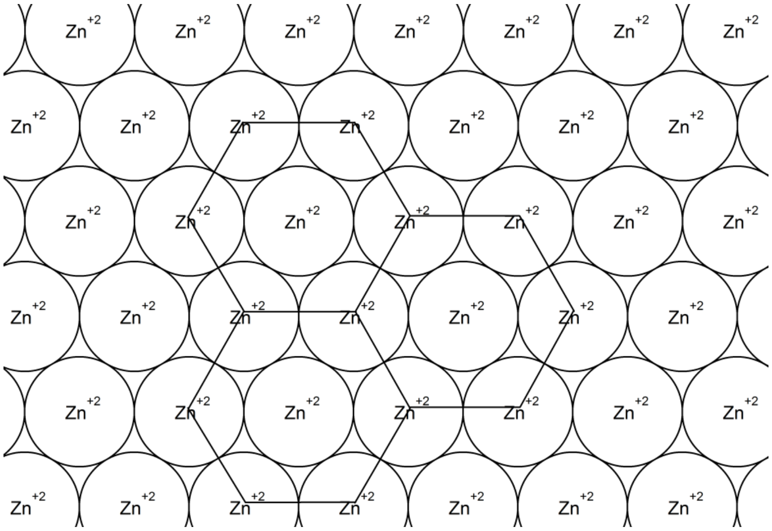

Supplementary Figure 4. | A 2D array of Zn2+ ions used to calculate the theoretical maximum 2D charge density, σ^{S}_{Max}, in order to check the charge density we obtained from Supplementary Eq. 7 is physically possible.

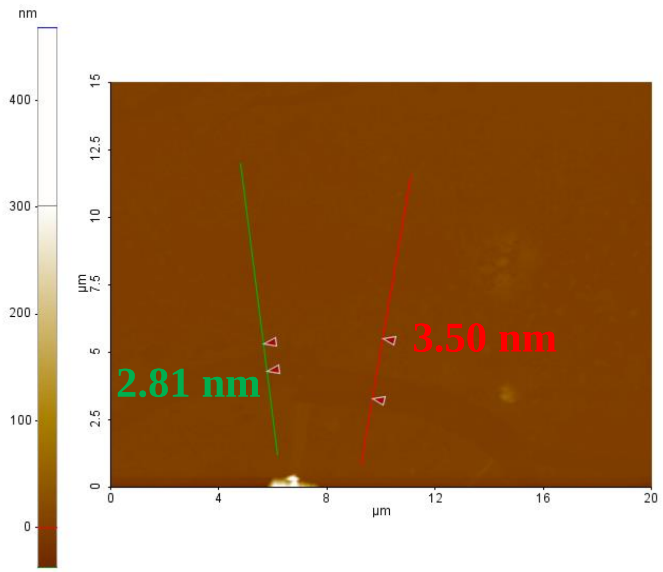

Supplementary Figure 5. | AFM topography scans to obtain the statistical measurement of the thickness of amorphous nanosheets. There were cracks in the millimeter-sized amorphous films from which thickness measurements can be done by topography scans.

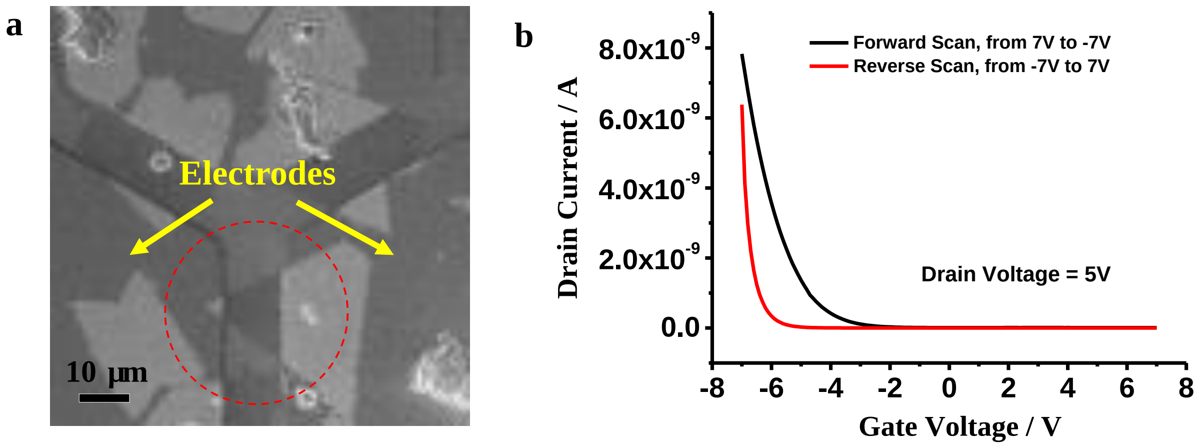

Supplementary Figure 6. | Further information on ZnO nanosheet FET devices. a. SEM image of the device (red dash circled) whose I-V characteristics were shown in the main text Figure 4. The metal pad array was defined by a TEM grid with ZnO nanosheets transferred directly from the surface of synthesis solution. The substrate was highly-doped Si wafer coated with 50 nm Al_2O_3 by atomic layer deposition. Some of the nanosheets fell right between two metal pads and hence formed FET devices. **b,** Forward and reverse scan of drain current *versus* gate voltage of the FET.

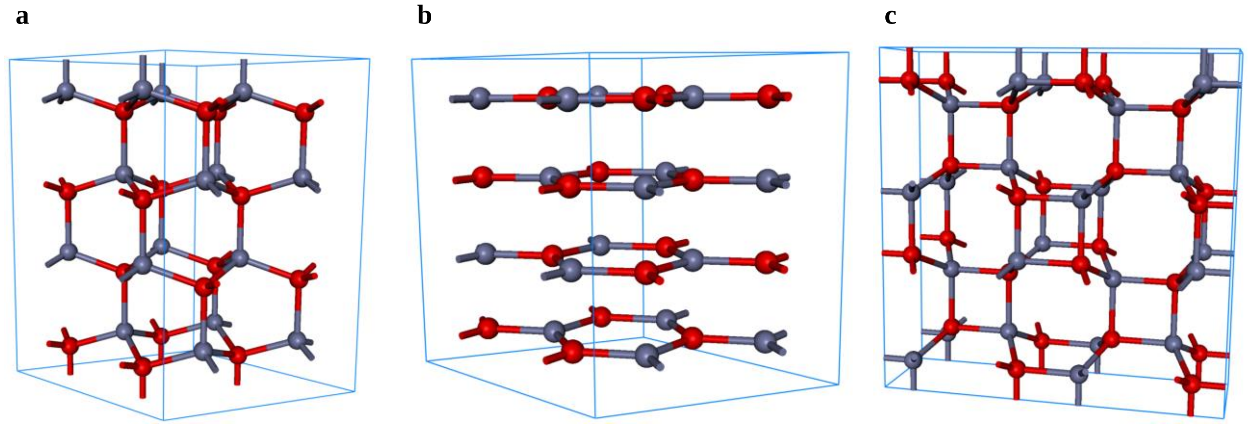

Supplementary Figure 7. | Crystal structures of a, Hexagonal Wurtzite ZnO, **b,** Planar ZnO, and **c,** tetragonal ZnO.

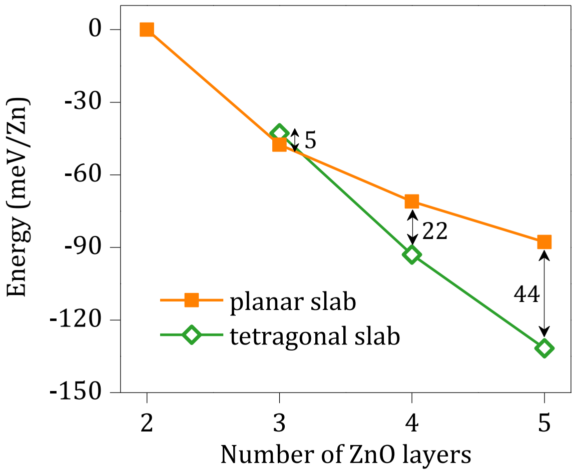

Supplementary Figure 8. | Total energy of bare planar and tetragonal Zn(0001) slabs with different thickness. The value of a 2-layer planar slab is set to zero. The 2-layer tetragonal slab is unstable and evolves to the planar structure during geometry optimization.

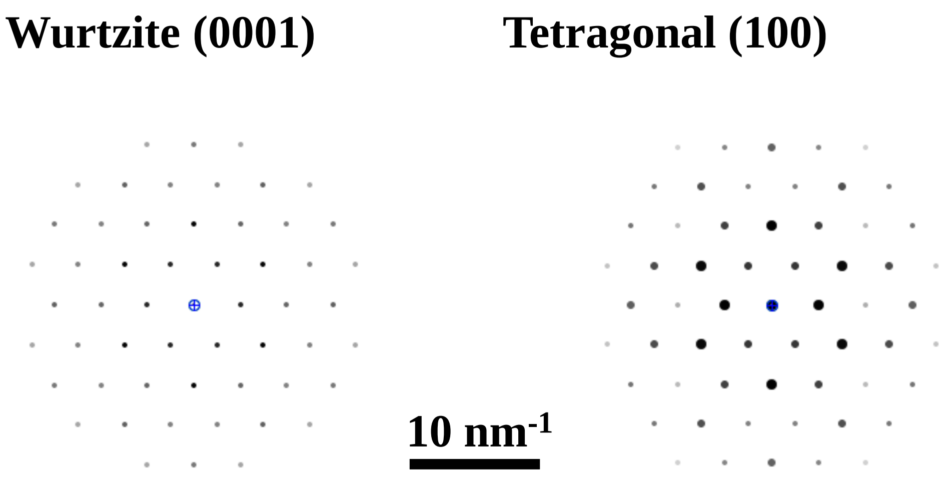

Supplementary Figure 9. | Simulated electron diffraction pattern along the surface normal of the nanosheets. Left, Hexagonal Wurtzite structure when the surface normal is along the *c*-axis of the lattice. **Right**, Tetragonal structure (P42/MNM) when the surface normal is along the *a*-axis of the lattice. The two diffraction patterns overlap with each other.

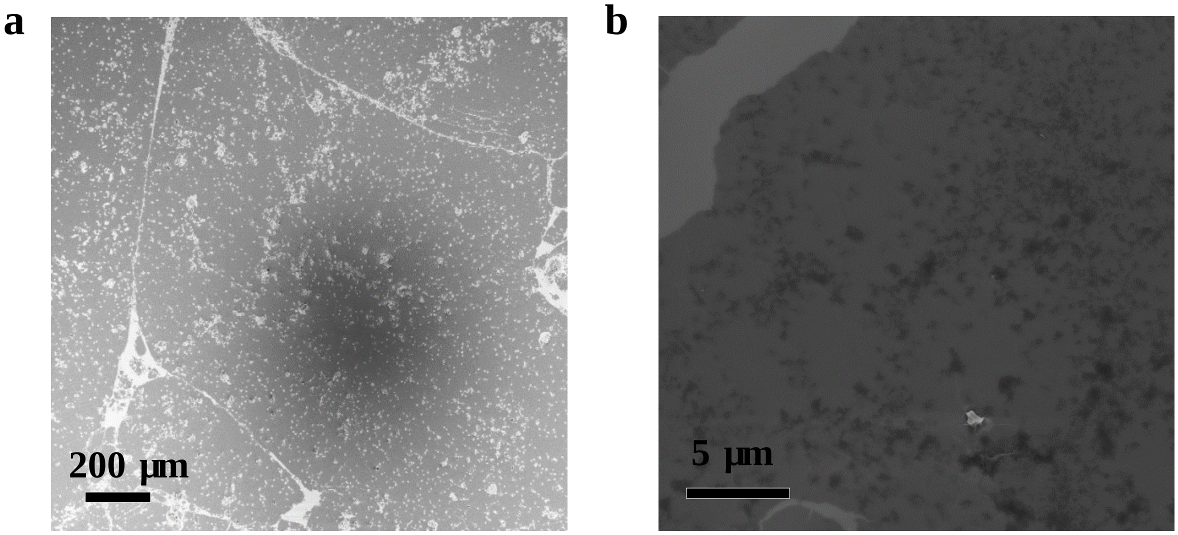

Supplementary Figure 10. | SEM images of nanosheets obtained when stearic acid was used to form the surfactant monolayer. **a,** A low-magnification image showing their sizes up to millimeters. **b,** A zoomed-in image showing the non-faceted edges of these nanosheets.

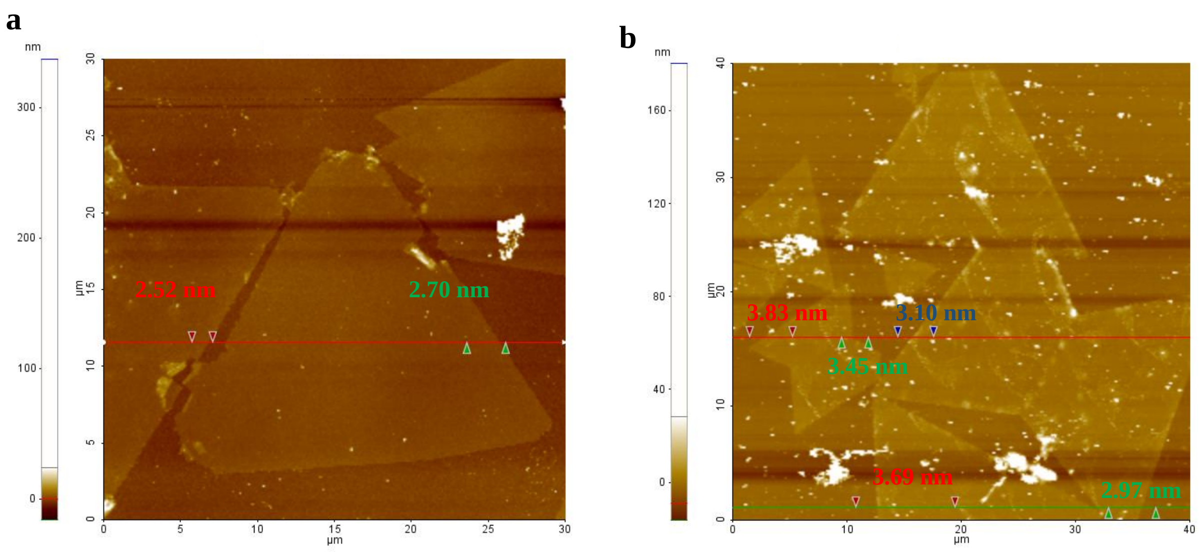

Supplementary Figure 11. | Examples of the measurement of the thickness of the nanosheets grown at a, 1.5X and b, 2X density of oleylsulfate monolayer by AFM.

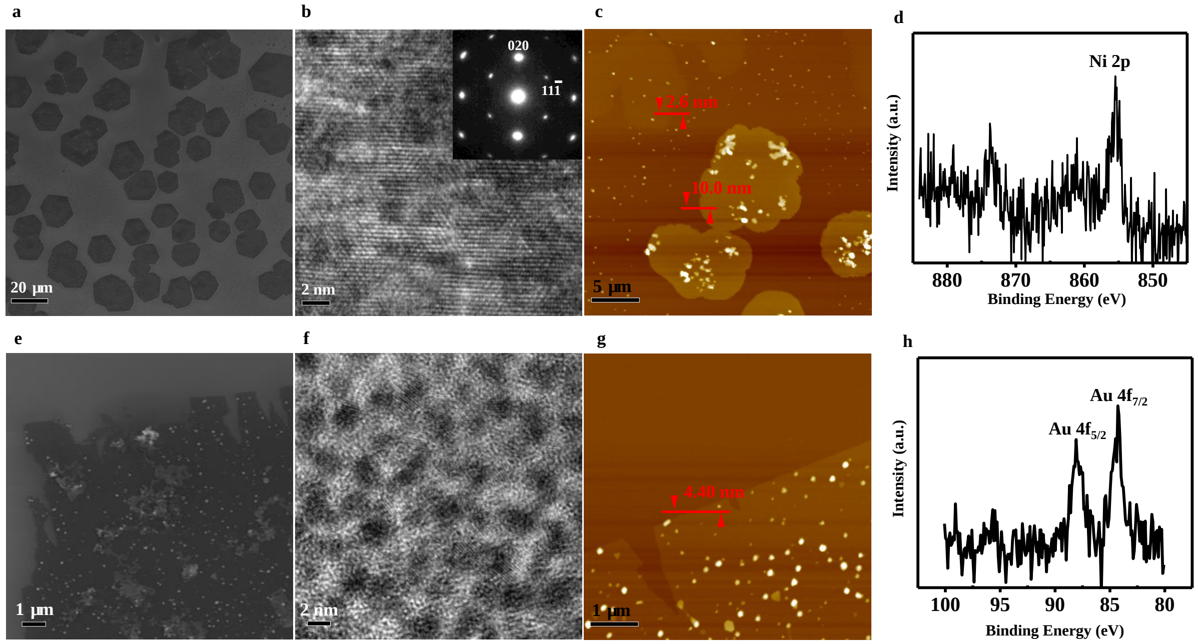

Supplementary Figure 12. | Structural characterization of NiO and Au nanosheets a and e, SEM images of NiO and Au nanosheets, respectively, **b and f,** high-resolution TEM images of NiO and Au nanosheets, respectively, **c and g,** AFM topography scans of NiO nanosheets and a Au nanosheet. **d and h,** XPS spectra of NiO nanosheets and Au nanosheets revealing the +2 and 0 valence states of Ni and Au, respectively.

Supplementary Tables

Species i	n_i^0 ($\frac{ions}{m^3}$)	z_i	$\phi_{x=0}$ (V)	n_i($\frac{ions}{m^3}$)
Zn^{2+}	1.12734×10^{25}	2	0.23309	1.27136×10^{32}
H^+	1.51269×10^{21}	1	0.23309	5.07991×10^{24}
OH^-	2.39746×10^{18}	-1	0.23309	7.13914×10^{14}
NO_3^-	2.93399×10^{25}	-1	0.23309	8.73681×10^{21}
NH_4^+	6.02063×10^{24}	1	0.23309	2.02184×10^{28}
$ZnNO_3^+$	7.70834×10^{23}	1	0.23309	1.27158×10^{27}

Supplementary Table 1. | Parameters used and calculated concentrations of various ions from Supplementary Eq. 2 assuming no physical size of ions.

	Wurtzite	Planar	Tetragonal
Bare ZnO(001) slab	unstable	44	0
Zn(0001) slab with surfactant	10	unstable	0

Supplementary Table 2. | Summary of the total energies (meV/Zn) of the three phases. Values of the tetragonal structures are set to zero.

Supplementary Notes

Supplementary Note 1: X-ray photoelectron spectroscopy of ZnO nanosheets

Supplementary Figure 2a, b, and c show the binding energy of Zn, and the Auger electron peaks of Zn and S respectively. The red curve in C was a smoothed version of the original spectrum. Supplementary Figure 2d is an SEM image of the focused area. The nanosheet in focus was on a metal pad on a Si/Al_2O_3 substrate. Supplementary Figure 3e is a Wagner plot showing the modified Auger parameter of Zn, which is the sum of the kinetic energy of the Auger transition and Zn $2p^{3/2}$ binding energy). From the plot we can see that the Zn^{2+} ions in ZnO nanosheets have the same modified Auger parameter as $ZnSO_4$. At the same time, their position on the Wagner plot is very close to that of organic Zn compound. Therefore we can infer that there was specific bonding between Zn^{2+} ions in ZnO nanosheets and the sulfate group in oleylsulfate surfactant. The position of Zn^{2+} ions in the Wagner plot is also within the range of ZnO.

Supplementary Note 2: DFT simulation methods and results on the crystal structure and band structure of ZnO nanosheets

For the bare ZnO(0001) slab, we surprisingly find that the most stable atomic arrangement is the tetragonal structure. In contrast, the planar structure is 44 meV per Zn atom (44 meV/Zn) higher than the tetragonal one. Although a bilayer planar ZnO(0001) was experimentally obtained on Ag(111),[1] our calculations suggest that the planar slab is more stable than the tetragonal one only when the thickness is equal to or less than three ZnO layers (Supplementary Figure 8). The wurtzite structure is the least stable form and it dynamically evolves to the planar structure in a fully geometry relaxation, which may explain the previous theoretical findings of planar structures.[2] For a ZnO(0001) slab with surfactants closely packed on one surface, the tetragonal structure is still the most stable one. The wurtzite-like structure is less stable than the tetragonal structure by 10 meV/Zn, while the planar structure becomes dynamically unstable. The energetics of the three phases are summarized in Supplementary Table 2.

Supplementary Note 3: Control experiments using stearic acid as the surfactant monolayer.

To further prove the critical role of the interactions between oleylsulfate and Zn^{2+} ions, we did the syntheses with the same experimental parameters except using stearic acid to replace oleylsulfate. As shown in Supplementary Figure 10, we could only obtain large area amorphous film with sporadic, small crystallites, without forming faceted triangular morphology, much like the amorphous film formed

initially during the synthesis using oleylsurfate. Therefore, it is certain that the interactions between oleylsulfate and Zn^{2+} ions are more than just electrostatic, and the specific bonding between the two parties is necessary to help the inter-distance between oleylsulfate molecules adapt to the Zn^{2+} ions in order to foster the epitaxial growth of ZnO nanosheets.

Supplementary Note 4: Thickness measurement of ZnO nanosheets at 1X, 1.5X, and 2X density oleylsulfate monolayers

To investigate how the density of surfactant monolayer may affect the thickness of the single-crystalline ZnO nanosheets, we conducted control experiments at 1X, 1.5X, and 2X density of oleylsulfate monolayers while keeping other experimental parameters the same. We observed that single-crystalline, triangular ZnO nanosheets with similar lateral sizes were formed in all experiments. This further supports our idea that the growth of single-crystalline ZnO nanosheets occurred in a different way from traditional epitaxy where the lattice parameters of the substrate (the surfactant monolayer in our case here) must match the growing material. Our ionic layer epitaxy growth is an adaptive epitaxy.

At the same time, we observed that the thickness of the ZnO nanosheets varies with different density of surfactant monolayer. At 1X, 1.5X, and 2X density of oleylsulfate monolayer, we surveyed the thickness of a number of nanosheets by AFM and obtained 2.84 ± 0.26 nm, 2.88 ± 0.41 nm, and 3.04 ± 0.48 nm. Supplementary Figure 11a and b show typical measurement of the thickness of ZnO nanosheets grown at 1.5X and 2X density of oleylsulfate monolayers, respectively.

Supplementary Note 5: Synthesis of NiO and Au nanosheets by ionic layer epitaxy

Supplementary Figure 12a shows an SEM image of the NiO hexagonal nanosheets. Similar to ZnO nanosheets, they are about 10 to 20 μm in length on their edges. Supplementary Figure 12b is a high-resolution TEM image taken from one of the hexagonal nanosheet. The TEM image reveals a single-crystalline rock salt structure of NiO and the exposed surface is (101). The inset is an electron diffraction pattern, in which the diffraction spots are indexed. Supplementary Figure 12c is a topography scan of a hexagonal NiO nanosheet and its thickness is found to be 10 nm or below. X-ray photoelectron spectrum (Supplementary Figure 12d) shows a Ni 2p peak where the binding energy is typically associated with Ni^{2+}. Supplementary Figure 12e is an SEM image of a corner of a sub-millimeter sized Au nanosheet. TEM characterization of this nanosheet (Supplementary Figure 12f) shows a polycrystalline nature with 2-4 nm crystalline size. AFM topography scan shows a thickness of 4.4 nm, which indicates the Au

nanosheet is a single layer of 2-D aggregation of Au nanoparticles. XPS spectrum (Supplementary Figure 12h) reveals the spin-orbital split peaks of Au 4f that are associated with metal Au.

Supplementary Discussions

We think the formation of the nanoparticles in the SEM images is likely due to the residue chloroform that was used to disperse the surfactant monolayer. The precursors might have gone into the organic phase where they nucleate and grow in a dissimilar way from the aqueous phase. There are two experiment evidences to support this. First, if we waited 5 to 10 minutes for the chloroform to completely evaporate before moving the glass vial to the oven to initiate the reaction, the amount of unwanted particles on the nanosheets were significantly reduced. Second, instead of a water-air interface, we are able to grow ZnO nanosheets at water-cyclohexane interface (Supplementary Figure 1d). In this case, none of the nanoparticles appeared on the nanosheets.

Supplementary Methods

X-ray photoelectron spectroscopy

XPS spectra of ZnO nanosheets were acquired by Thermo K-alpha X-ray photoelectron spectrometer with a focused, monochromatic X-ray source and monoatomic ion gun.

Calculation of electric potential profile and Zn^{2+} concentration profile from the surfactant-water interface into the bulk solution.

A charged surface, such as the surfactant monolayer, will push and pull on charged species in solution. The ions in solution will reorganize themselves about the charged surface, screening monolayer's influence on the bulk solution. The ratio of concentrations of species i in bulk solution (n_i^0) at bulk solution potential (φ^0 =0) to its concentration (n_i) found at any other potential (φ) is taken to depend upon the Boltzmann factor in the following way:

$$n_i = n_i^0 e^{\left(\frac{-z_i e \phi(x)}{kT}\right)} \tag{1}$$

where z_i is the charge of ion *i*, *e* is the charge of the electron, φ is the potential relative to bulk solution, *x* is a measure of distance into solution and perpendicular to the monolayer surface, *k* is the Boltzmann constant, and *T* is absolute temperature. Under meager potentials, e.g. 1 Volt, and reasonable bulk ion concentrations, Supplementary Eq. 1 will produce large, nonphysical ion concentrations (e.g. $n_i > \frac{1\ \text{Mole}}{\text{cm}^3}$). To determine whether 'large' potentials would be found in the studied system, Supplementary Eq. 2 and Supplementary Eq. 3 were used.[3]

$$\sigma^{M} = \epsilon\epsilon_0 \left(\frac{d\phi}{dx}\right)_{x=0} \tag{2}$$

$$\left(\frac{d\phi}{dx}\right) = \sqrt{\frac{2kT}{\epsilon\epsilon_0}\sum_i \left(n_i^0\left[e^{\left(\frac{-z_i e\phi_{x=0}}{kT}\right)} - 1\right]\right)} \tag{3}$$

where σ^{M} is the charge density of the monolayer, ϵ is the relative permittivity of the material (80 for water), ϵ_0 is the electrical permittivity of free space, $\left(\frac{d\phi}{dx}\right)_{x=0}$ is the potential gradient (electric field strength) at the monolayer's surface, and $\phi_{x=0}$ is the potential at the monolayer's surface. Taking σ^{M} as -0.801 C m^{-2} (i.e. $\frac{1\ \text{electron}}{20Å^2}$) and solving for $\phi_{x=0}$, we indeed encountered large potentials that were capable of inducing large, nonphysical ion concentrations. Supplementary Table 1 shows the type of ion concentrations expected from applying Supplementary Eq. 2 directly to the experimental conditions we studied.

If we model the hydrated ions as hard spheres, then the maximum packing density possible is the close packing structure. Taking Zn^{2+} ions as an example, each with a hydration diameter (d) of 6 Å,[4] then the maximum 3D packing density possible is given by:

$$\text{Maximum 3D Density} = \frac{Zn^{+2}\ \text{ions}}{\text{Volume}} = \frac{\left(\frac{3}{2}\right)+3+\left(\frac{3}{2}\right)}{\frac{3\sqrt{3}}{2}a^2c} = \frac{6}{\frac{3\sqrt{3}}{2}d^2\left(2d\sqrt{\frac{2}{3}}\right)} = 6.54729 * 10^{27}\,\frac{\text{ions}}{m^3} \tag{4}$$

Thus, the total of all ion concentrations at the closest approach to the 2D charged layer must be equal to or less than this atomic density.

$$\sum n_i \leq 6.54729 * 10^{27}\,\frac{\text{ions}}{m^3} \tag{5}$$

Supplementary Table 1 shows a clear violation of this condition.

Iterative numerical calculation was applied to find the limit of ϕ at which Supplementary Eq. 2 and Supplementary Eq. 3 obtained the condition required in Supplementary Eq. 5. This yielding the critical

criteria for the surface potential: $\phi_{x=0} \leq 0.090994$ V. In order to achieve this potential, far less than the 0.23309 V previously determined to be present at the charged monolayer, a charged stern layer is assumed to form adjacent to the charged self-assembled surfactant monolayer. Using Supplementary Eq. 2 and Supplementary Eq. 3 again, except now fixing $\phi_{x=0} = 0.090994$ V and solving for σ, it is found that a surface charge density of $0.0651358\frac{C}{m^2}$ is capable of maintaining a $\phi_{x=0} = 0.090994$ V in the required aqueous conditions. Physically, this apparent surface charge density σ^A is the 2D charge density necessary to create an electric field strong enough to condense the charged species in solution into a close packing structure. Because the superposition principle applies in electromagnetism, the 2D charge density (σ^A) that the solution experiences is a sum of the surfactant monolayer's charge density (σ^M) and the Stern layer's charge density (σ^S).

$$\sigma^A = \sigma^M + \sigma^S \tag{6}$$

To gain insight into the nature of the charged Stern layer, and determine if it is physical reasonable, rearranging Supplementary Eq. 7 and solving for σ^S yields:

$$\sigma^S = \sigma^A - \sigma^M = (-0.0651358) - (-0.801) = 0.735952\frac{C}{m^2} \tag{7}$$

This value for σ^S is well within the theoretical maximum 2D charge density (σ^S_{Max}) obtained by modeling the Stern layer as a 2D array of charged Zn^{+2} ions (Supplementary Figure 4).

The following is used to calculate σ^S_{Max}:

$$\sigma^S_{Max} = \frac{\text{Charge}}{\text{Area}} = \frac{\left(\frac{\text{Zn}^{+2}\text{ ions}}{\text{hexagon}}\right)\text{x}\left(\frac{\text{Charge}}{\text{Zn}^{+2}\text{ ion}}\right)}{\frac{3\sqrt{3}}{2}\text{d}} = \frac{\left(6\left(\frac{1}{3}\right)+1\right)\text{x}(2*\text{e})}{\frac{3\sqrt{3}}{2}(\text{d})} = 1.0278\frac{C}{m^2} \tag{8}$$

σ^S_{Max} is greater than the previously determined value of $0.735952\frac{C}{m^2}$ for σ^S and thus σ^S is deemed a physically reasonable value for the stern layer charge density.

ZnO-based FET and calcations on the carrier concentration and hole mobility

Supplementary Figure 6a shows a metal pad array with nanosheets on them and Supplementary Figure 6b shows the single nanosheet-based FET from which the I-V characteristics in main text Figure 4 were obtained.

The hole concentration and hole mobility were calculated by following two equations, respectively.

$$n_e = \frac{V_{th} \cdot C_{ox}}{q \cdot w \cdot L}, \ C_{ox} = \frac{A_{channel} \cdot \varepsilon_0 \cdot \varepsilon_{ox}}{t_{ox}}, \quad (9)$$

and $$\mu = \frac{L^2 \cdot g_m}{C_{ox} \cdot V_{ds}}, \quad (10)$$

where V_{th} is the threshold voltage. C_{ox} is the capacitance of the dielectric oxide. Q is the charge of an electron. W, h, and L are the channel width, height, and length of the nanosheet, respectively. $A_{channel}$ is the channel area defined by the channel length times channel width. ε_0 and ε_{ox} are the electrical permittivity of vacuum and Al_2O_3, respectively. The latter was assumed to be 10 based on literature value and the growth condition.[5,6] t_{ox} is the thickness of Al_2O_3. g_m is the transconductance at gate voltage V_{ds}.

DFT simulation on the crystal structure and band structure of ZnO nanosheets

We performed density functional theory calculations with the Vienna *ab initio* simulation package.[7,8] The Perdew–Burke–Ernzerhof (PBE) exchange-correlation functional was employed except the band structure calculations, where the Heyd-Scuseria-Ernzerhof (HSE06) functional was used instead to obtain more accurate results.[9] The projector augmented wave method was used together with the following potentials: H_GW ($1s^1$, E_{cut} = 300.0 eV), C_GW_new ($2s^2 2p^2$, E_{cut} = 414.0 eV), O_s_GW ($2s^2 2p^4$, E_{cut} = 334.7 eV), S_GW ($3s^2 3p^4$, E_{cut} = 258.7 eV), and Zn_pv_GW ($3d^{10} 4s^2$, E_{cut} = 360.2 eV). The plane-wave energy cutoff was set to 500 eV. The ZnO(0001) slab consists of a 2 × 2 supercell (~6.5 Å ×6.5 Å) in plane and five bulk ZnO atomic layers together with a ~10 Å of vacuum layer out of plane. The Brillouin zone is sampled with a 5 × 5 × 1 Monkhorst-Pack grid. The atom positions and in-plane lattices are fully relaxed until the force on each is less than 10^{-3} eV/Å. The surfactant molecules are packed closely on the slab surface, with a density of one surfactant molecule per four surface Zn atoms. To reduce the computational cost, a 3-carbon chain is used to represent the 18-carbon chain in oleylsulfate. Tests show that this simplification has a minor influence to the properties we are interested in.

Simulated electron diffraction pattern of ZnO nanosheets

JEMS was used to simulate the electron diffraction patterns along the surface normal of the nanosheets for Wurtzite structure (0001) and the tetragonal structure (100), as shown in Supplementary Figure 9.

Synthesis of NiO nanosheets by ionic layer epitaxy

In a typical synthesis of NiO nanosheets, 0.0648g $NiCl_2$ was dissolved in 50 mL DI water. After complete dissolution, 130 μL hydrazine was added to the aqueous solution. Subsequently, 10 mM 1M NaOH solution was added, yielding a clear green solution. Depending on the opening size of the reactor, 0.02 mg/L chloroform solution of stearic acid was spread onto the surface of the growth solution. After 5 mins, the solution was placed in 70 ℃ oven and the reaction was conducted for 30 minutes.

Synthesis of Au nanosheets by ionic layer epitaxy

To synthesis Au nanosheets, in a 6-dram vial, 10μL of a 0.10 M NaOH solution was diluted with 5.790mL of water and 1.000mL of a 2.0 mM L-Arginine solution. To this, 0.200mL of 17.14mM HAuCl4 solution was added and gently swirled to combine. 10μL of stearic acid in chloroform (1mg/5mL) was gently dropped on top of the water and left to evaporate for 10 minutes. The vial was then capped tightly and placed in a 90 ℃ convection oven for 2 to 7 hours. The vial was left to cool to room temperature and the surface of the water was then sampled with a SiO_2-coated silicon wafer for SEM imaging.

Supplementary References

1 Tusche, C., Meyerheim, H. L. & Kirschner, J. Observation of depolarized ZnO(0001) monolayers: Formation of unreconstructed planar sheets. *Phys. Rev. Lett.* **99** (2007).

2 Freeman, C. L., Claeyssens, F., Allan, N. L. & Harding, J. H. Graphitic nanofilms as precursors to wurtzite films: Theory. *Phys. Rev. Lett.* **96** (2006).

3 Bard, A. J. & Faulkner, L. R. *Electrochemical Methods: Fundamentals and Applications*. (Wiley, 2000).

4 Persson, I. Hydrated metal ions in aqueous solution: How regular are their structures? *Pure Appl. Chem.* **82**, 1901-1917 (2010).

5 Tanner, C. M., Perng, Y. C., Frewin, C., Saddow, S. E. & Chang, J. P. Electrical performance of Al_2O_3 gate dielectric films deposited by atomic layer deposition on 4H-SiC. *Appl. Phys. Lett.* **91** (2007).

6 Yota, J., Shen, H. & Ramanathan, R. Characterization of atomic layer deposition HfO_2, Al_2O_3, and plasma-enhanced chemical vapor deposition Si_3N_4 as metal-insulator-metal capacitor dielectric for GaAs HBT technology. *J. Vac. Sci. Technol., A* **31**, 01A134 (2013).

7 Kresse, G. & Furthmuller, J. Efficiency of ab-initio total energy calculations for metals and semiconductors using a plane-wave basis set. *Comp Mater Sci* **6**, 15-50 (1996).

8 Kresse, G. & Furthmuller, J. Efficient iterative schemes for ab initio total-energy calculations using a plane-wave basis set. *Phys. Rev. B* **54**, 11169-11186 (1996).

9 Heyd, J., Scuseria, G. E. & Ernzerhof, M. Hybrid functionals based on a screened Coulomb potential. *J. Chem. Phys.* **118**, 8207-8215 (2003).
